# Reproductive and sexual health in the Maldives: analysis of data from two cross-sectional surveys

**DOI:** 10.1186/1472-6963-11-S2-S6

**Published:** 2011-12-21

**Authors:** Anne Cockcroft, LuWei Pearson, Candyce Hamel, Neil Andersson

**Affiliations:** 1 CIET Trust Botswana, PO Box 1240, Gaborone, Botswana; 2 UNICEF Ethiopia, PO Box 1169, Addis Ababa, Ethiopia; 3 CIETcanada, 1 Stewart Street, Ottawa, Ontario, Canada; 4 Centro de Investigación de Enfermedades Tropicales, Universidad Autónoma de Guerrero, Acapulco, México

## Abstract

**Background:**

The Maldives faces challenges in the provision of health services to its population scattered across many small islands. The government commissioned two separate reproductive health surveys, in 1999 and 2004, to inform their efforts to improve reproductive and sexual health services.

**Methods:**

A stratified random sample of islands provided the study base for a cluster survey in 1999 and a follow-up of the same clusters in 2004. In 1999 the household survey enquired about relevant knowledge, attitudes and practices and views and experience of available reproductive health services, with a focus on women aged 15-49 years. The 2004 household survey included some of the same questions as in 1999, and also sought views of men aged 15-64 years. A separate survey about sexual and reproductive health covered 1141 unmarried youth aged 15-24 years.

**Results:**

There were 4087 household respondents in 1999 and 4102 in 2004. The contraceptive prevalence rate (CPR) for modern methods was 33% in 1999 and 34% in 2004. Antenatal care improved: more women in 2004 than in 1999 had at least four antenatal care visits (90.0% v 65.1%) and took iron supplements (86.7% v 49.6%) during their last pregnancy. The response rate for the youth survey was only 42% (varying from 100% in some islands to 12% in sites in the capital). The youth respondents had some knowledge gaps (one third did not know if people with HIV could look healthy and less than half thought condoms could protect against HIV), and some unhelpful attitudes about gender and reproductive health.

**Conclusions:**

The two household surveys were commissioned as separate entities, with different priorities and data capture methods, rather than being undertaken as a specific research study. The direct comparisons we could make indicated an unchanged CPR and improvements in antenatal care, with the Maldives ahead of the South Asia region for antenatal care. The low response rate in the youth survey limited interpretation of the findings. But the survey highlighted areas requiring attention. Surveys not undertaken primarily for research purposes have important limitations but can provide useful information.

## Background

The Republic of Maldives has 199 inhabited islands and about 1000 other islands, including resorts, stretching north-south across 500 miles of the Indian Ocean. These islands form 26 natural atolls, which are grouped into 20 administrative units. Most of the islands are small, no island is more than eight Km long, and all are low lying with an average elevation of 1.6m above sea level. The geography of the country, coupled with the costs of transport and diseconomies of scale, makes delivery of public services of any kind very expensive and difficult. This is certainly true of reproductive health services, especially obstetric care.

The population of the Maldives was 270,101 in 2000 [1]. In addition, there were 19,000 resident foreign workers and their dependants. About 27% of the population lived in the capital island of Maleˊ. Maleˊ also has a floating population of several thousands who come from other islands for commerce, education and medical treatment.

In 1999, the government of the Maldives commissioned a national reproductive health survey to provide information about relevant knowledge, attitudes and practices of the population (particularly women aged 15-49 years) and their experience and views of available reproductive health services [2]. The survey was intended to provide a picture of the current situation and to inform implementation of a five year reproductive health programme (1998-2002) [3]. Five years later, in 2004, the government commissioned another reproductive health survey [4] to inform ongoing reproductive and sexual health programmes, supported by the UNFPA in the Maldives [5]. In this survey, the Ministry of Health (MoH) wanted more information from men, now recognised as being important to involve in reproductive health programmes [6]. Also, by this time, the country's reproductive and sexual health programme had a particular focus on adolescents [5] and so an additional survey of unmarried youth was commissioned. The surveys were commissioned under two separate contracts. For the 1999 survey, CIET (Centro de Investigación de Enfermedades Tropicales; Community Information and Epidemiological Technologies) was commissioned to provide technical support for survey design, implementation, analysis, and reporting. The Health Information and Research Unit (HIRU) of the MoH was responsible for data collection and data entry but CIET provided skilled personnel to train and supervise field workers, and to supervise data entry. In 2004, the HIRU was fully responsible for the surveys, including training and supervision for field work and data entry, and CIET was contracted only to provide technical advice. In neither contract was there any budget or time allocated for capacity building but it was hoped that CIET researchers would impart skills to personnel from the HIRU and MoH while working with them.

This article describes the reproductive health survey methods, which differed between 1999 and 2004, and the changes in those indicators that could be compared directly between 1999 and 2004. It also describes the methods and findings of the survey of unmarried youth in 2004. We discuss the challenges and limitations of the work, and the extent to which these affect the interpretation of the findings.

## Methods

### The sample

The 1999 survey used a multi-stage cluster sample drawn to be representative of the national population. We stratified the islands into six regions, and then divided each region into three groups: those with a regional hospital (one such island per region); those with a health centre; and those without either. We randomly selected islands within each stratum, the sampling fraction being chosen to give a sample size in each stratum proportional to the relative population in that stratum. For most of the selected islands, the total number of households was less than 150 and the sample site included all the households on the island (although not all the households actually participated in the survey). For larger islands, an area sample, randomly selected from a plan of the island, of approximately 100 households was the sample site. The five sites in Male' each consisted of three contiguous enumeration blocks and were selected randomly from among enumeration blocks in the 1995 census.

The 2004 survey revisited the same sample sites as in 1999. For most islands in the sample this meant the same households were included in 1999 and 2004, but we did not follow-up individual households between surveys. In the 2004 survey, interviewers did not collect data from more than 100 households in any site.

The HIRU contacted island administrators and community leaders in the selected islands ahead of the visits of the field teams, to explain the survey and secure their support during data collection.

### The questionnaires

The 1999 household questionnaire, developed in consultation with the MoH and other stakeholders, focused on knowledge and practices about contraception, knowledge about sexually transmitted diseases and AIDS, antenatal care, and experience of reproductive health services. Respondents were women in the household aged 15-49 years and the male head of the household. Interviewers asked respondents the questions and recorded their responses, including responses to a number of open-ended questions, in data registers (“Bhopal books” [7,8]) with the questionnaire attached inside the front and back covers and responses for each respondent recorded on a separate page of the book.

In 2004, interviewers administered the household questionnaire to all ever-married women aged 15-49 years and all ever-married men aged 15-64 years in the household. The questionnaire was prepared in a scannable format, with the interviewers shading bubbles to indicate responses; nearly all questions had response options rather than being open-ended. The design team focused on the information aims of the 2004 survey and did not make particular efforts to ensure questions were comparable with those in the 1999 survey.

In 2004, another questionnaire was used to collect information from unmarried youth (aged 15-24 years) identified in the households. The interviewers invited eligible youth to attend an “event” at a venue in or near the site, giving them a numbered ticket to hand in at the event. The details of the event varied between sites but it was generally a gathering held in the evening, with food, soft drinks, and music provided. The youth who attended completed an anonymous self-administered questionnaire, in a scannable format, in the presence of a facilitator who could clarify any points about the questions. The questionnaire covered the youth's age, sex, education, financial status, their use of TV, radio, internet and written materials, their knowledge about sexually transmitted infections and AIDS, sources of information about sex, sexual activity, experience of forced sex, condom use, and pregnancies.

### Data management and analysis

In 1999, computer operators manually entered the data from the data registers; double data entry with validation using Epi Info software [9] minimised keystroke errors and further checking identified inconsistent and out of range responses. In 2004, trained operators scanned the completed questionnaires using Remark software [10].

The original analysis of the 1999 and 2004 datasets relied on Epi Info software. We calculated weights to allow for some oversampling of some strata in the household sample and reported on weighted percentages of indicators. In practice, the weighted and unweighted values were very similar. We did not attempt to weight the youth sample. Details of the values of all indicators from the 1999 and 2004 surveys can be found in the individual reports [2,4].

For the purposes of this article, we analysed the two surveys as being a repeated cross-sectional study design. The analysis reported here relied on CIETmap open source software [11]. We express the significance of differences between 1999 and 2004 using the Mantel Haenszel Odds Ratio (OR) [12] and the 95% confidence interval (CI). We adjusted the CI for clustering within sites (CIca), using a method described by Gilles Lamothe [13], based on a variance estimator to weight the Mantel Haenszel Odds Ratio for cluster-correlated data [14,15].

## Results

The 1999 household survey included 4,087 respondents from 2,254 households, while that in 2004 included 4,102 respondents from 2,279 households. The field teams did not collect data on the number of households and individuals that were approached and declined to participate in the survey, nor on the number of households where they found no one at home at the time of the survey. Anecdotal evidence from the supervisors suggests that few of the eligible people present when the interviewers came to the door refused to participate (L Pearson, personal communication). Table 1 shows basic characteristics of the respondents in 1999 and 2004. In 1999, the survey included women aged 15-49 years old in the households, together with male household heads. The household head was male in 52.9% of the households (1,193/2,254). The 2004 survey included ever-married women aged 15-49 years and ever-married men aged 15-64 years. In order to allow like-with-like comparisons between 1999 and 2004 responses, we compared the responses of ever-married women in 1999 with ever married women in 2004, and compared ever-married men in 1999 (the number was small as it only included household heads) with the larger number of ever-married men aged 15-64 interviewed in 2004 (Table 1).

**Table 1:** Characteristics of the respondents in the two surveys

	1999	2004
**Household respondents**		
Number of households	2254	2279
Females 15-49	3193	
Ever married females 15-49	2439^a^	2693
Males 15-64 (household heads)	447	
Ever married males 15-64	429^b^	1409
**Youth respondents** **^c^**		
Unmarried females 15-24	-	663
Unmarried males 15-24	-	469

^a^ In 1999, interviewers spoke to all women aged 15-49 in the households; in 2004, they only interviewed ever married females aged 15-49

^b^ In 1999, interviewers only spoke to men who were the head of the household; in 2004, they included all ever married men aged 15-64

^c^ The survey of unmarried youth was only included in 2004

Table 2 summarises the comparison of reproductive health indicators between 1999 and 2004, among ever-married women aged 15-49 years. There was no significant change in overall contraceptive prevalence rate between 1999 and 2004, and the CPR for modern methods was 30.9% in 1999 and 32.5% in 2004 (difference not significant at the 5% level). In 2004, more women had antenatal care than in 1999, considering either any visits or considering the recommended four visits (Table 2). Similarly, more women in 2004 than in 1999 took iron supplements in their last pregnancy, whether considering any supplements or considering supplements for at least four months (Table 2). There was a higher proportion of women with secondary education in the 2004 survey than in the 1999 survey. Taking this into account by stratification did not explain the higher proportions of pregnant women using antenatal care and taking iron in 2004.

**Table 2:** Reproductive health indicators among ever-married women aged 15-49 years in 1999 and 2004

Indicator	% (fraction)		OR (95% CIca)^1^
	1999	2004	
Contraceptive prevalence (all methods)	39.5 (937/2372)	37.6 (1000/2662)	0.92 (0.77-1.10)
Contraceptive prevalence (modern methods)	30.9 (734/2372)	32.5 (864/2662)	1.07 (0.89-1.29)
Rated reproductive health services as “good”	86.7 (2493/2875)	80.7 (2033/2518)	0.64 (0.44-0.94)
Any antenatal visits in last pregnancy^2^	89.4 (1981/2215)	99.6 (273/274)	32.3 (4.38-237.44)
Four or more antenatal visits in last pregnancy^2^	65.1 (1443/2215)	90.0 (249/274)	5.33 (3.42-8.31)
Took iron in last pregnancy^2^	49.6 (1075/2169)	86.7 (222/256)	6.64 (4.24-10.42)
Took iron for at least 4 months in last pregnancy^2^	23.7 (515/2169)	63.7 (163/256)	5.63 (3.74-8.47)

^1^ Odds Ratio comparing 2004 with 1999; with cluster-adjusted 95% confidence interval

^2^ In 2004, only pregnancies during the last 12 months were included

A total of 1,141 youth (unmarried young men and women aged 15-24 years old) completed a questionnaire. Only 42% of eligible youth identified in the households participated in the survey; the response rate ranged from 100% in some islands to only 12% for sites in Maleˊ. More than one half (58.6%) of the 1,132 youth who completed a questionnaire and gave information about their sex were female. Table 3 summarises some of the findings from the youth survey, among the youth who completed a questionnaire. More than half were currently in school and about half reported they had money available to spend on themselves. Almost all had heard of HIV/AIDS. But their knowledge was lacking in some areas; less than half knew that one can avoid catching HIV by always using a condom, and less than half knew that people with HIV or AIDS can look healthy. Less than half thought “boys and girls are equal”, but most believed it was not necessary to have sex with a boyfriend or girlfriend to show love. A sizeable minority, especially among young men (30%), thought talking about condoms would make young people more promiscuous. Less than half said they talked to anyone about sex. Few admitted they had already had sex: 14% of boys and 5% of girls. Nearly as many boys (3%-4%) as girls (4%-5%) said they had been involved in unwanted sex. Among girls, nearly as many as admitted to having sex at all said someone had involved them in unwanted sex. Few youth (2.6% of females, 1.7% of males) admitted to a pregnancy or having fathered a child.

**Table 3:** Findings among unmarried youth aged 15-24 years who completed a questionnaire

Indicator	% (fraction)	
	Males	Females
Currently at school	60.6 (283/467)	59.0 (388/658)
Have money to spend on themselves	55.7 (244/438)	52.9 (274/618)
Have heard of HIV/AIDS	95.5 (448/469)	97.9 (641/655)
Know that can avoid catching HIV by always using a condom	45.8 (215/469)	42.5 (282/663)
Know that people with HIV/AIDS can look healthy	45.7 (207/453)	45.5 (290/637)
Think “boys and girls are equal”	45.8 (206/450)	49.1 (309/629)
Think “you don’t need to have sex with your boyfriend/girlfriend to show that you love them”	77.4 (342/442)	77.5 (496/640)
Believe that talking about condoms makes young people more promiscuous	30.3 (135/445)	19.2 (122/637)
Talk with anyone about sex	46.8 (217/464)	38.7 (254/657)
Ever had sex	13.7 (63/459)	5.2 (34/653)
Someone their age^1^ involved them in unwanted sex	3.7 (17/458)	4.0 (26/656)
Someone older^2^ involved them in unwanted sex	3.3 (15/455)	5.0 (33/654)
Ever been pregnant or know have fathered a child	1.7 (8/459)	2.6 (17/658)

^1^ Within five years of their own age

^2^ Older by five years or more

## Discussion

### Limitations

There are a number of limitations to the surveys and the analysis described in this article. Lack of a control group limits the conclusions one can draw about attribution of any changes over time to a particular programme or intervention. This is a problem common to most evaluations of national programmes. There are possible ways to overcome this, provided there is forward planning and strong government commitment, such as undertaking a stepped-wedge trial, a type of pragmatic randomised cluster controlled trial [16]. Future CIET social audits in the health sector are exploring such design options [17].

The fact that the surveys were not undertaken as a research project, but rather as two separately commissioned surveys, led to a number of difficulties in making direct comparisons between the two time points. The second survey was designed in a scannable format mainly because the MoH HIRU wanted experience in handling data obtained using scannable instruments. A secondary reason was that it had proved difficult to identify and train local data entry operators for the 1999 survey, given the small pool of people with suitable basic skills for this task. Problems with the quality of data entry in 1999 meant some parts of the dataset had to be re-entered. The 2004 scannable format meant modifications to questions, with loss of comparability with questions in the 1999 survey. Shifting priorities of the MoH meant a focus on a slightly different group of respondents in 2004, which meant we had to restrict our analysis to comparable subsets rather than simply compare the whole sample in 1999 with that in 2004. In the end, we were able to make some defensible comparisons between 1999 and 2004, helped by the fact that the teams re-visited the same sites in 2004, hence reducing sampling errors. However, we were only able to compare some outcomes.

The lack of a formal recording of response rates in the household survey was an omission in both 1999 and 2004. Although we believe the non-response rates were low, we do not have formal evidence of this. If the non-response rates were relatively high, this could have biased the findings in the individual surveys, and if they differed between the two surveys, this could have affected the changes we found in some indicators.

We were able to measure response rates in the youth survey by comparing the number of “tickets” given to youth identified in the household survey with the number of youth who completed a questionnaire. The response rates were low, mainly due to very low response rates in sites in Maleˊ and near to Maleˊ. This could have biased the results and certainly limits the extent to which they can be generalised. The Maleˊ sites included many youth living away from their original homes, in order to study or work in Maleˊ. These youth, who may have different knowledge, attitudes and practice compared with youth living at home, are seriously under-represented in the sample. We do not know the reasons why so many youth did not participate. The decision not to interview young people in their home setting was reasonable, given the need to secure privacy when answering sensitive questions about sexual behaviour. The strategy of bringing them to a central place also meant the questionnaires could be self-administered (but with a facilitator present), again increasing the likelihood of disclosure of sensitive information. Sensitivity of the topic is a possible reason for the overall low response rate, but the high response rate in some sites makes this unlikely to be the main reason. It is possible that some of the field teams did not make strong enough efforts to encourage youth to attend the event where they completed questionnaires: perhaps the event was not interesting enough, or not well publicised. If this was the reason, it may not have introduced much bias. On the other hand, youth who participated might have been more compliant and less rebellious than those who did not, tending to bias the findings. Overall, the youth survey probably underestimated risky sexual attitudes and behaviour among youth. Despite this, it did indicate some areas of concern about knowledge and attitudes and was therefore at least a useful pointer for programme focus areas.

A declared aim of both surveys was local capacity building. Yet capacity building opportunities were limited by lack of budget and time for any such activities. On-the-job learning is important but not enough and this was a source of frustration. Current CIET social audits and those planned in the future include a much greater element of funded capacity building [18].

### Reproductive health survey findings

The 1999 survey was the first to produce national figures for reproductive health indicators in the Maldives. We shared the findings with government and non government stakeholders concerned with reproductive health as an input into the implementation of the national reproductive health programme. In 1999, the Maldives had relatively low rates of contraception compared with other countries in South Asia at about the same time. The CPR in the Maldives, for all methods and for modern methods, was lower than that in Bangladesh [19] and India [20], similar to in Nepal [21], and higher than that in Pakistan [22]. There was little change in the CPR in the Maldives between 1999 and 2004 (Table 2); in a similar period the CPR increased in Nepal [23], Bangladesh [24], and India [25], while in Pakistan the low CPR remained unchanged [26]. The CPR in the Maldives in 2009 measured in the Demographic and Health Survey (DHS) [27], was no higher than in our measurement in 2004. Clearly, further efforts are needed if the use of contraception in the Maldives is to increase.

On the other hand, the Maldives compares favourably with other countries in South Asia for the proportion of women attending for antenatal care. The 1999 figure for any antenatal care in the Maldives was substantially higher than in Nepal, Bangladesh, India, or Pakistan [19-22], and the situation improved further so that by 2004 virtually all pregnant women in the Maldives had at least one antenatal care visit and nine out of ten had four visits or more, making it the leader in the region [15-18]. The high rates of antenatal care were confirmed in the 2009 DHS [27]. The proportion of women taking iron during pregnancy is one indicator of the quality of antenatal care. This figure in the Maldives in 1999 was quite similar to other countries in South Asia [19-22], but by 2004 the proportion of pregnant women taking iron in the Maldives was well above that in other countries [23-26], and the high figure has been maintained in 2009 [27]. The relatively good antenatal care in the Maldives has been achieved despite the considerable logistic difficulties of providing services to the scattered islands. Estimated maternal mortality has fallen much more rapidly in the Maldives between 1990 and 2008 than in other countries in the region and the 2008 figure (37) is almost an order of magnitude less than the regional average (280) [28].

### Youth survey findings

As discussed above, the low response rate means that only tentative conclusions can be drawn from the youth survey. The findings of the 2009 DHS in the Maldives [27], although the young women the DHS interviewed were “ever married” rather than unmarried, suggests some of the 2004 youth survey findings may have been a reasonable reflection of reality. In both the 2004 survey and the 2009 DHS, virtually all the respondents had heard of HIV/AIDS. The reported proportions of youth aged 15-24 who had heard of AIDS in other countries in the region were somewhat lower, especially among young women [23-26]. Less than half the 2004 respondents agreed that always using a condom was a way to avoid catching HIV; the proportion knowing condom use was a way to avoid infection was higher in the DHS, undertaken five years later [27].

In countries of sub-Saharan Africa at the centre of the AIDS epidemic, gender based violence is an important driver of the epidemic, particularly the high rates of infection among young women [29]. It is important to understand the views and experience of youth about gender roles and gender based violence in countries such as the Maldives, in order to inform efforts to avoid an epidemic similar to that in southern Africa. The 2004 survey of sexual health among adolescents was a start in this direction. More studies of the views and experience of youth are needed, as well as trials of interventions to support youth and reduce gender based violence.

### Lessons learned

Several useful lessons emerged from the experience of analysing the findings from the surveys described here. It is not uncommon for repeat surveys in the same place to be separately contracted and with differing priorities. At least in this case both the surveys were undertaken by the same organisation, giving an opportunity to merge datasets and make direct comparisons over time. In a similar situation in the future, we would emphasize more strongly the benefits of being able to make direct comparisons over time and try to agree with the commissioning entity a core set of questions to be included in both surveys. It is hard to criticise the decision to switch to a scannable format in the second household survey in order to build local capacities in this technology; perhaps both surveys should have used this data capture method. In retrospect, we should have insisted on piloting the method of inviting youth to an event to complete their questionnaires; one should not assume that what sounds like a good idea will actually work well in practice. The lack of a control group for the before-and-after design is a universal problem when evaluating national programmes. Methods to overcome it, such as stepped-wedge trials, require substantial forward planning and commitment from governments. Giving government technical advisers and policy makers an orientation about the requirements for evidence based planning could help. This is included in our current programme of capacity building related to HIV trials in Southern Africa [30,31]. In the surveys in the Maldives, the commissioning bodies had limited funding available and, as is very often the case, this meant there was no funding available to fulfil the worthy aspiration for capacity building. Perhaps when negotiating the details of a contract, the implementing organisation should insist on either (preferably) including a realistic budget and time allowance for capacity building or removing reference to it altogether.

## Conclusions

The two household surveys were commissioned as separate entities, with different priorities and data capture methods, rather than being undertaken as a specific research study. Only limited direct comparisons over time could be made. Nevertheless, these indicated an unchanged CPR and improvements in antenatal care, with the Maldives ahead of the region for antenatal care. The low response rate in the youth survey limited interpretation of the findings. Nonetheless, the survey highlighted areas requiring attention to support youth and reduce risks of rising HIV rates. Surveys not undertaken primarily for research purposes have important limitations but can provide useful information.

## List of abbreviations used

AIDS: Acquired Immune Deficiency Syndrome; HIRU: Health Information and Research Unit of the Ministry of Health; HIV: Human Immunodeficiency Virus; MoH: Ministry of Health of government of Maldives; UNFPA: United Nations Population Fund

## Competing interests

The authors declare they have no competing interests.

## Authors' contributions

AC supported the design, analysis and reporting of the surveys. She undertook the analysis for this paper and drafted the manuscript. LP assisted with the baseline survey design and implementation, the analysis and reporting, and helped to finalise the manuscript. CH prepared the datasets for the analysis in this paper and helped to finalise the manuscript. NA provided technical support for the design and analysis and helped to finalise the manuscript.

## References

[B1] Ministry of Planning and National Development, Maldives Population and housing census of Maldives 2000 2001 Male' http://www.planning.gov.mv/publications/Pop_housing_census2000/

[B2] Pearson L Cockcroft A Maldives reproductive health survey, 1999 1999 Male', Maldives: Ministry of Health http://www.ciet.org/_documents/2006224101536.pdf

[B3] Executive board of the United Nations Development Programme and of the United Nations Population Fund United Nations Population Fund, country programme for Maldives 1998-2002 http://countryoffice.unfpa.org/maldives/drive/CPIIFinalVersion_dpfmamdv2.pdf

[B4] Merhi S Cockcroft A Andersson N Maldives reproductive health survey 2004 2004 Submitted to UNFPA and MoH Maldives http://www.ciet.org/_documents/2006224101944.pdf

[B5] Executive board of the United Nations Development Programme and of the United Nations Population Fund United Nations Population Fund, country programme for Maldives 2003-2007 http://countryoffice.unfpa.org/maldives/drive/CPIIIFinalVersion_dpfpamdv3.pdf

[B6] Sternberg P Hubley J Evaluating men's involvement as a strategy in sexual and reproductive health promotion Health Promot Int 2004 19 389 396 10.1093/heapro/dah312 15306623

[B7] Andersson N Muir MK Mehra V Bhopal eye Lancet 1984 2 1481 6151098 10.1016/s0140-6736(84)91685-4

[B8] Andersson N Ajwani MK Mahashabde S Tiwari MK Muir MK Mehra V Ashiru K Mackenzie CD Delayed eye and other consequences from exposure to methyl isocyanate: 93% follow up of exposed and unexposed cohorts in Bhopal Br J Ind Med 1990 47 553 558 2393636 10.1136/oem.47.8.553 PMC1035230

[B9] Centers for Disease Control and Prevention: Epi Info Version 6. Public domain softwarehttp://www.cdc.gov/epiinfo

[B10] Remark optical mark recognition (OMR) softwarehttp://www.gravic.com/remark/officeomr/

[B11] Andersson N Mitchell S Epidemiological geomatics in evaluation of mine risk education in Afghanistan: introducing population weighted raster maps Int J Health Geogr 2006 5 1 10.1186/1476-072X-5-1 16390549 PMC1352365

[B12] Mantel N Haenszel W Statistical aspects of the analysis of data from retrospective studies of disease J Natl Cancer Inst 1959 22 719 748 13655060

[B13] Andersson N Lamothe G Clustering and meso-level variables in cross sectional surveys: an example of food aid during the Bosnian crisis. BMC Health Serv Res 2011 11 Suppl 2 S15 22376353 10.1186/1472-6963-11-S2-S15 PMC3332559

[B14] Bieler GS Williams RL Cluster sampling techniques in quantal response teratology and developmental toxicity studies Biometrics 1995 51 764 776 10.2307/2532963 7662858

[B15] Williams RL A note on robust variance estimation for cluster-correlated data Biometrics 2000 56 645 646 10.1111/j.0006-341X.2000.00645.x 10877330

[B16] Brown CA Lilford RJ The stepped wedge trial design: a systematic review BMC Med Res Methodol 2006 6 54 10.1186/1471-2288-6-54 17092344 PMC1636652

[B17] Andersson N Proof of impact and pipeline planning: directions and challenges for social audit in the health sector BMC Health Services Research 2011 11 Suppl 2 S16 22376386 10.1186/1472-6963-11-S2-S16 PMC3332560

[B18] Andersson N Building the community voice into planning: 25 years of methods development in social audit BMC Health Services Research 2011 11 Suppl 2 S1 22376121 10.1186/1472-6963-11-S2-S1 PMC3397387

[B19] National Institute of Population Research and Training (NIPORT) Mitra and Associates (MA) ORC Macro (ORCM) Bangladesh Demographic and Health Survey 1999-2000 2001 Dhaka, Bangladesh and Calverton, Maryland: NIPORT, MA, and ORCM

[B20] International Institute Population Sciences (IIPS) ORC Macro National Family Health Survey (NFHS-2), 1998-99: India 2000 Mumbai: IIPS

[B21] Ministry of Health [Nepal] New ERA ORC Macro Nepal Demographic and Health Survey 2001 2002 Calverton, Maryland, USA: Family Health Division, Ministry of Health; New ERA; and ORC Macro

[B22] National Institute of Population Studies (NIPS) [Pakistan] Pakistan Reproductive Health and Family Planning Survey 2000-01 2001 Islamabad, Pakistan: National Institute of Population Studies

[B23] Ministry of Health and Population (MOHP) [Nepal] New ERA Macro International Inc Nepal Demographic and Health Survey 2006 2007 Kathmandu, Nepal: Ministry of Health and Population, New ERA, and Macro International Inc

[B24] National Institute of Population Research and Training (NIPORT) Mitra and Associates ORC Macro Bangladesh Demographic and Health Survey 2004 2005 Dhaka, Bangladesh and Calverton, Maryland [USA]: National Institute of Population Research and Training, Mitra and Associates, and ORC Macro

[B25] International Institute for Population Sciences (IIPS) Macro International National Family Health Survey (NFHS-3), 2005–06: India: Volume I 2007 Mumbai: IIPS

[B26] National Institute of Population Studies (NIPS) [Pakistan] Macro International Inc Pakistan Demographic and Health Survey 2006-07 2008 Islamabad, Pakistan: National Institute of Population Studies and Macro International Inc

[B27] Ministry of Health and Family (MOHF) [Maldives] ICF Macro Maldives Demographic and Health Survey 2009 2010 Calverton, Maryland: MOHF and ICF Macro

[B28] World Health Organisation Trends in maternal mortality: 1990 to 2008 2010 Geneva http://whqlibdoc.who.int/publications/2010/9789241500265_eng.pdf

[B29] Andersson N Cockcroft A Shea B Gender-based violence and HIV: relevance for HIV prevention in hyperendemic countries of southern Africa AIDS 2008 22 Suppl 4 S73 S86 10.1097/01.aids.0000341778.73038.86 19033757

[B30] Andersson N Cockcroft A Shea B Thabane L Who should drive solutions to the AIDS epidemic in southern Africa: lessons from the African Development of AIDS Prevention Trials capacity (ADAPT) network AIDS Impact Conference, Gaborone, Botswana, 22-25 Sept 2009 Abstract 220. http://www.aidsimpact.com/2009/Academics/Programme/abstract/?id=220

[B31] Global Health Research Initiative African Development of AIDS Prevention Trials capacity (ADAPT2) http://www.idrc.ca/EN/Programs/Global_Health_Policy/Global_Health_Research_Initiative/Pages/ProjectDetails.aspx?ProjectNumber=106354

